# Supplementary material for: RAL GTPases mediate EGFR-driven intestinal stem cell proliferation and tumourigenesis
Source: eLife. 2021 Jun 7;10:e63807. doi: 10.7554/eLife.63807 (PMC8216719; doi:10.7554/eLife.63807)
Supplement: Transparent reporting form [file elife-63807-transrepform.docx]

***eLife’s* transparent reporting form**

We encourage authors to provide detailed information *within their submission* to facilitate the interpretation and replication of experiments. Authors can upload supporting documentation to indicate the use of appropriate reporting guidelines for health-related research (see [EQUATOR Network](http://www.equator-network.org/%20)), life science research (see the [BioSharing Information Resource](https://biosharing.org/)), or the [ARRIVE guidelines](http://www.plosbiology.org/article/info:doi/10.1371/journal.pbio.1000412) for reporting work involving animal research. Where applicable, authors should refer to any relevant reporting standards documents in this form.

If you have any questions, please consult our Journal Policies and/or contact us: [editorial@elifesciences.org](mailto:editorial@elifesciences.org).

**Sample-size estimation**

- You should state whether an appropriate sample size was computed when the study was being designed
- You should state the statistical method of sample size computation and any required assumptions
- If no explicit power analysis was used, you should describe how you decided what sample (replicate) size (number) to use

Please outline where this information can be found within the submission (e.g., sections or figure legends), or explain why this information doesn’t apply to your submission:

No formal calculation was performed to determine sample size. Sample size was decided empirically based on publishing standards and experience with the inherent variability of particular experiments.

**Replicates**

- You should report how often each experiment was performed
- You should include a definition of biological versus technical replication
- The data obtained should be provided and sufficient information should be provided to indicate the number of independent biological and/or technical replicates
- If you encountered any outliers, you should describe how these were handled
- Criteria for exclusion/inclusion of data should be clearly stated
- High-throughput sequence data should be uploaded before submission, with a private link for reviewers provided (these are available from both GEO and ArrayExpress)

Please outline where this information can be found within the submission (e.g., sections or figure legends), or explain why this information doesn’t apply to your submission:

Information regarding the number of replicates is available on the figures themselves with details of what n represents in the figure legend.

All experiments represent at least two independent biological replicates. Each biological replicate for *Drosophila* experiments represent a set of control and experimental tissues (specific genotypes indicated in Supplementary Table 1) collected and processed from independent crosses on different days and reared on independent batches of standard medium. Additionally, key experiments were replicated using independent genetic constructs. These are provided as figure supplements.

For cell culture experiments each biological replicate represents an independent transfection with the relevant RNAi vector. Technical replicates for the in vitro internalization assay are repeat measurements using samples from the same cell culture dish.

RNASeq data is available through GEO.

**Statistical reporting**

- Statistical analysis methods should be described and justified
- Raw data should be presented in figures whenever informative to do so (typically when N per group is less than 10)
- For each experiment, you should identify the statistical tests used, exact values of N, definitions of center, methods of multiple test correction, and dispersion and precision measures (e.g., mean, median, SD, SEM, confidence intervals; and, for the major substantive results, a measure of effect size (e.g., Pearson's r, Cohen's d)
- Report exact p-values wherever possible alongside the summary statistics and 95% confidence intervals. These should be reported for all key questions and not only when the p-value is less than 0.05.

Please outline where this information can be found within the submission (e.g., sections or figure legends), or explain why this information doesn’t apply to your submission:

Key statistical methods and what n and error bars represent are included in each figure legend. Statistical significance is indicated on figures as follows: *p<0.05, **p<0.01, ***p<0.001, ****p<0.0001.

In graphs where multiple comparisons were performed, only statistical findings directly related to the points being delt with in the results section where indicated. This was due to space restrictions within the graphs and to make data interpretation more amenable to the reader.

Individual data points are presented for image quantifications but not for pH3 quantification due to the often-high n number in the latter and narrow graphs.

(For large datasets, or papers with a very large number of statistical tests, you may upload a single table file with tests, Ns, etc., with reference to sections in the manuscript.)

**Group allocation**

- Indicate how samples were allocated into experimental groups (in the case of clinical studies, please specify allocation to treatment method); if randomization was used, please also state if restricted randomization was applied
- Indicate if masking was used during group allocation, data collection and/or data analysis

Please outline where this information can be found within the submission (e.g., sections or figure legends), or explain why this information doesn’t apply to your submission:

In midgut regeneration experiments animals from the same tube were randomly allocated into vials containing either 5% sucrose or Ecc15 in 5% sucrose-soaked filter paper.

**Additional data files (“source data”)**

- We encourage you to upload relevant additional data files, such as numerical data that are represented as a graph in a figure, or as a summary table
- Where provided, these should be in the most useful format, and they can be uploaded as “Source data” files linked to a main figure or table
- Include model definition files including the full list of parameters used
- Include code used for data analysis (e.g., R, MatLab)
- Avoid stating that data files are “available upon request”

Please indicate the figures or tables for which source data files have been provided:

Sequencing data is available through the Gene Expression Omnibus (GEO) repository. All remaining raw data pertaining to the final manuscript are publicly available through the University of Glasgow's repository <http://dx.doi.org/10.5525/gla.researchdata.1142>. Code used for data analysis is freely available through GitHub.
